# A Terrible Disease

**Published:** 1888-02

**Authors:** 


A Terrible Disease.—A colored clergyman once preached a sermon on the text: "And the multitudes came to Him, and He healed them of divers diseases." Said he: "My dying congregation, this is a terrible text. Disease is in the world. The small-pox slays its hundreds, the cholera its thousands, and the yellow fever its tens of thousands, but, in the language of the text, if you take the divers, you are gone. These earthly doctors can cure the small-pox, cholera, and the yellow fever if they get there in time, but nobody but the good Lord can cure the divers."

